# Effect of complete reduction of hernia sac and transection of hernia sac during laparoscopic indirect inguinal hernia repair on seroma

**DOI:** 10.1186/s12893-022-01599-8

**Published:** 2022-04-25

**Authors:** Chunpeng Pan, Xin Xu, Xianke Si, Jiwei Yu

**Affiliations:** 1 Department of General Surgery, Shanghai Ninth People’s Hospital, School of Medicine, Shanghai Jiao Tong University, 639 Zhizaoju Road, Huangpu, Shanghai, 200011 China; 2 Department of Minimally Invasive Surgery, Putuo Hospital Affiliated to Shanghai University of Traditional Chinese Medicine, 164 Lanxi Road, Putuo, Shanghai, 200062 China

**Keywords:** Laparoscopic, Transection, Reduction, Hernia sac, Seroma

## Abstract

**Introduction:**

This study investigated the effect of complete reduction and transection of the hernia sac during laparoscopic indirect inguinal hernia repair on seroma.

**Methods:**

Retrospective analysis was performed on 1763 cases undergoing laparoscopic indirect inguinal hernia repair in three centers from January 2017 to September 2019, among them, 311 patients with transection of hernia sac and 1452 patients with reduction of hernia sac, the data of the two groups were tested by t-test. Logistic univariate analysis was performed on 233 cases of postoperative seroma, and variables p < 0.05 in univariate analysis were included for multivariate analysis. Then, the transection group and the reduction group were matched with 1:1 propensity score matching, and the caliper value was set at 0.05. Finally, 274 patients matched in each group were analyzed by univariate analysis again to evaluate whether the transection of hernia sac had an impact on postoperative seroma.

**Results:**

The results of univariate analysis of 233 patients with postoperative seroma showed that: ASA-3 p = 0.031, classification-L3 p < 0.001, surgery-TEP p < 0.001, transect group p = 0.005. The results of multivariate analysis show that: ASA-3 p < 0.001, classification-L3 p < 0.001, surgery-TEP p < 0.001, transect group p = 0.020. The results of univariate analysis after propensity score matching showed that transection of the hernia sac is significant for postoperative seroma (p < 0.001).

**Conclusion:**

Transection of the hernia sac during laparoscopic indirect inguinal hernia repair can significantly lead to postoperative seroma.

**Supplementary Information:**

The online version contains supplementary material available at 10.1186/s12893-022-01599-8.

## Introduction

An inguinal hernia is a protrusion of abdominal tissue from a defect in the abdominal wall to the body surface. It is a common disease in general surgery [1, 2]. There are more than 20 million cases of inguinal hernia repair worldwide every year [3, 4]. When patients with disease attack, abdominal pain is often severe accompanied by abdominal mass, or intestinal obstruction, electrolyte disorders, etc., causing serious impact on patients' life safety. The common approaches of hernia repair surgery include open tension-free hernia repair and laparoscopic hernia repair. Laparoscopic hernia repair has the advantages of quick postoperative recovery, low postoperative infection rate [5], short hospital stay [6, 7], mild postoperative pain [8, 9], low recurrence rate and good curative effect [10]. Laparoscopic hernia repair has become increasingly popular [11]. At present, transabdominal preperitoneal (TAPP) and totally extraperitoneal (TEP) are relatively common.

With the continuous application of laparoscopy in the field of hernia surgery, its advantages have been continuously proved, but the occurrence of postoperative seroma has always puzzled patients and surgeon, especially after laparoscopic indirect hernia repair. It has been reported that the incidence of postoperative seroma is 3.7 ~ 70%, but seroma can generally be absorbed within 3 months after surgery [12–14].Some scholars have found that the separation of mesh and surrounding tissue is also related to the occurrence of seroma [15].Therefore, it is great significance to find the related factors of seroma formation. The treatment of hernia sac is the most important step in the operation, which is related to the treatment and long-term effect of the operation [16], it is especially closely related to the occurrence of postoperative seroma. Seroma is the earliest and most common complication of laparoscopic inguinal hernia repair [17, 18], and it can mimic an early recurrence. A prospective study showed that the risk of postoperative seroma after transection of the inguinal hernia sac during laparoscopic hernia repair was higher than in patients with complete dissection of the sac [19]. The formation of seroma was closely related to the size of the hernia sac and the duration of operation. Because of the long course of disease, the hernia sac adhered to the surrounding tissues and was dense, the neck structure of the hernia sac was vague and fibrosis, and the spermatic cord was difficult to separate out, leading to severe exudation after surgery and easy formation of seroma [20]. In laparoscopic indirect hernia repair, there is still no agreement on which surgical method can reduce the occurrence of postoperative seroma, compared with the complete reduction and transection of the hernia sac [21].

This is a retrospective study and discussed the influence of complete reduction of hernia sac and transection of hernia sac during laparoscopic indirect inguinal hernia repair on seroma. It is hoped that the results of this study can provide some reference for surgeons in the treatment of hernia sac during laparoscopic hernia repair in the future.

## Materials and methods

### Objectives and study design

Seroma is defined as a fluid collection in the underlying tissue lacunae and in the lacunae formed after surgery due to aseptic inflammatory response and exudate accumulation. The diagnostic criteria for postoperative seroma is that the patient has a mass in the surgically treated side of the inguinal region and that there is effusion within the mass as confirmed by ultrasound (DW-500) or CT (German Siemens 64 row 128 slice CT machine). Sac volume was calculated using the formula for an ellipsoid [V = (4/3)πabc], where a, b, and c represent the radius of the length, width, and height, respectively).Fluid collection in > 10 ml in volume was interpreted as seroma-positive [22].

A total of 1763 patients undergoing laparoscopic inguinal hernia repair with complete case data from three clinical medical centers for hernia from January 2017 to January 2019 were included in this study. All relevant data were collected by the surgeon from the medical record system. Postoperative patients were followed up for 6 months. Data from these patients were statistically analyzed to determine the relationship between postoperative seroma and hernia sac management after laparoscopic indirect inguinal hernia surgery.

Included in the standard: Primary indirect inguinal hernia, and for EHS L1-3 type, the surgical method was TEP or TAPP. Exclusion standard: All types of hernia except indirect inguinal hernia, for example direct inguinal hernia, femoral hernia, recurrent hernia, incarcerated hernia, strangulated hernia and other hernia types were excluded, cases transferred to laparotomy were excluded, cases of emergency surgery were excluded.

### Surgical technique

All operations were performed by experienced, senior surgeons from the three clinical hernia centers, all operations were performed with three intraoperative trocar holes (the layout of the three holes in TAPP, 10 mm trocar was inserted below the umbilicus as the observation hole, and two 5 mm trocars were inserted at the intersection of the left and right midclavicular line and the horizontal line 2 cm below the umbilicus as the operation holes. In TEP, 10 mm trocar was inserted below the umbilicus as the observation hole, and two 5 mm trocars were inserted between the pubic tubercle and the umbilicus on the median ventral line respectively as the operation holes), the intraoperative pneumoperitoneum pressure was set as 12 mmHg, and the pneumoperitoneum flow was set as 10 L/min, no intraoperative complications occurred during the operation. The operative strategy was to perform complete reduction of the hernia sac, but transection of the sac was performed in cases with severe adhesions. In the reduction group, the sac was isolated from the vas deferens and testicular vessels, and then it was completely reduced. In the transection group, the sac was isolated from the vas deferens and testicular vessels in an identical manner and transected at the midportion. The hernia sac is treated with electrical instruments. All cases were completely retained the results of spermatic cord or round ligament of the uterus. The patch with lightweight patches of polypropylene materials, the flat cover every case in the myopectineal orifice. Its coverage as the medial border to the pubic symphysis to 1 cm, the bottom boundary comb to Cooper’s ligament. The outer border to the anterior superior spine, upper boundary to conjoined tendon. The mesh is UMM3 produced by Johnson Medical Equipment Co., LTD., the size of which is 15*15 cm. The mesh was fixated by permanent tacks or glue.

### Statistical analysis

SPSS version 23.0 (SPSS Inc., Chicago, IL, USA) software was used for data analysis. They were divided into two groups, one group of 1452 patients who had the hernia sac completely reduced during the operation, and the other group of 311 patients who had the hernia sac transected during the operation, Logistic univariate analysis was performed on 233 cases of postoperative seroma, and the variables p < 0.05 in univariate analysis were included for multivariate analysis, and P < 0.05 was considered statistically significant. In this study. Continuous variables were examined by independent sample t-test or Wilcoxon rank-sum test, and categorical variables were compared by Pearson chi-square test, Fisher’s exact test or CMH chi-square test as appropriate.

Subjects in transection group and reduction group likely differ for confounders and differences in outcomes can reflect differences in baseline conditions rather than a real outcome, to eliminate bias and maximize the quality of the evidence provided by the study, we used propensity score matching to match patients with the transection group and the reduction group on a 1:1 basis, and a total of 548 patients were included for subsequent analysis. Univariate analysis was performed on the two groups after propensity score matching, and P < 0.05 was considered statistically significant.

## Results

**Fig. 1:**
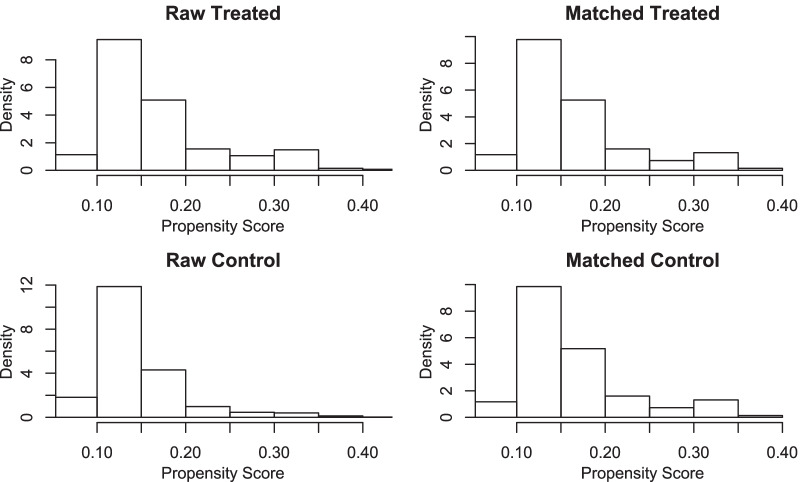
Matching effect display of propensity score. The propensity score was evenly distributed compared with raw control after matching

This study included 1763 patients from three clinical center since January 2017 to January 2019. All patients underwent laparoscopic inguinal hernia repair, according to whether the intraoperative transection hernial sac. It can be divided into two groups, 311 patients with transected hernia sac, 1452 patients with complete reduction of hernia sac. T-test was used to analyze the data of the two groups of patients. The results showed that there were significant differences between the two groups in sex (p < 0.001), and seroma (p = 0.011) (p < 0.05) (Table 1). Univariate analysis was performed on 233 patients with seroma, and then multivariate analysis was performed on factors (ASA-3 p = 0.031, classification-L3 p < 0.001, surgery-TEP p < 0.001, transect-yes p = 0.005) with p < 0.05. Finally, multivariate analysis showed that ASA-3 p < 0.001, classification-L3 p < 0.001, surgery-TEP p < 0.001, transect-yes p = 0.020 (Table 2). Because the baseline levels of the two groups(reduction of hernia sac group and transection of hernia sac group) were different, in order to exclude the influence of other factors on postoperative seroma other than the treatment of hernia sac, propensity score matching was performed at 1:1, and finally concluded that 274 patients in each group (Fig. 1, Additional file 1: Fig. S1). Univariate analysis was performed on the two groups after propensity score matching. The results showed that transect-yes p < 0.001 (Table 3).

**Table 1:** Baseline demographic characteristic

Variables	Total (n = 1763)	Non-transect (n = 1452)	Transect (n = 311)	p
*Sex, n (%)*				** < 0.001**
Female	150 (8)	106 (7)	44 (14)	
Male	1613 (92)	1346 (93)	267 (86)	
*Age (years old), n (%)*				0.261
< = 60	548 (31)	443 (30)	105 (34)	
> 60	1215 (69)	1009 (70)	206 (66)	
*BMI (kg/m*^*2*^*), n (%)*				0.650
< = 24	1131 (64)	928 (63)	203 (67)	
> 24	632 (36)	524 (37)	108 (33)	
ASA*, n (%)				0. 293
1	815 (46)	659 (45)	156 (50)	
2	863 (49)	723 (50)	140 (45)	
3	85 (5)	70 (5)	15 (5)	
*Position, n (%)*				0.806
Left	669 (38)	524 (37)	145 (39)	
Right	1094 (62)	873 (63)	221 (61)	
*EHS Classification, n (%)*				0.889
L1-2	1163 (66)	958 (66)	208 (67)	
L3	600 (34)	494 (34)	103 (33)	
*Length (cm), n (%)*				0.397
< = 1	42 (2)	31 (2)	11 (4)	
< = 3	669 (38)	553 (37)	116 (40)	
< = 4	828 (47)	708 (48)	120 (43)	
> 4	224 (13)	187 (13)	37 (13)	
*Seroma, n (%)*				**0.011**
No	1530 (87)	1274 (88)	256 (82)	
Yes	233 (13)	178 (12)	55 (18)	

^*^ASA, American society of anesthesiologists; Length, The length of the hernia sac,which is the distance from the deep inguinal ring to the bottom of the hernia sac

**Table 2:** Logistic univariate analysis and multivariate analysis for the risk factors of postoperation-seroma

Variables	Univariate analysis		Multivariate analysis	
	OR* (95% CI*)	p	OR (95% CI)	p
*Sex, n (%)*				
Female	Reference			
Male	3.992 (0.973–6.914)	0.051		
*Age(years old), n (%)*				
< = 60	Reference			
> 60	1.111 (0.791–1.586)	0.551		
*BMI(kg/m*^*2*^*), n (%)*				
< = 24	Reference			
> 24	1.219 (0.938–1.657)	0.201		
ASA*, n (%)				
1	Reference		Reference	
2	1.845 (0.716–4.753)	0.205	1.131 (0.629–1.878)	0.133
3	2.786 (1.098–7.068)	**0.031**	1.652 (1.153–3.198)	** < 0.001**
*Position, n (%)*				
left	Reference		Reference	
right	0.735 (0.477–1.133)	0.163	1.102 (0.748–1.641)	0.198
*EHS Classification, n (%)*				
L1-2	Reference		Reference	
L3	0.418 (0.278–0.610)	** < 0.001**	0.458 (0.301–0.678)	** < 0.001**
*Surgery, n (%)*				
TAPP	Reference		Reference	
TEP	0.615 (0.461–0.820)	** < 0.001**	0.519 (0.373–0.720)	** < 0.001**
*Length (cm), n (%)*				
< = 1	Reference			
< = 3	2.544 (0.621–4.218)	0.093		
< = 4	2.382 (0.673–4.692)	0.097		
> 4	2.531 (0.753–5.021)	0.079		
*Transect, n (%)*				
No	Reference		Reference	
Yes	1.566 (1.120–3.138)	**0.005**	1.603 (1.065–2.366)	**0.020**

^*^OR, odd ratio; 95% CI, 95% confidence intervals; ASA, American society of anesthesiologists. Length, The length of the hernia sac,which is the distance from the deep inguinal ring to the bottom of the hernia sac

**Table 3:** Logistic univariate analysis after propensity score matching

Variables	Univariate analysis	
	OR* (95% CI*)	p
*Sex, n (%)*		
Female	Reference	
Male	2.375 (0.671–6.473)	0.194
*Age(years old), n (%)*		
< = 60	Reference	
> 60	1.172 (0.503–1.994)	0.873
*BMI (kg/m*^*2*^*), n (%)*		
< = 24	Reference	
> 24	0.508 (0.250–0.962)	0.047
ASA*, n (%)		
1	Reference	
2	1.021 (0.05–6.218)	0.986
3	2.449 (1.338–4.703)	**0.005**
*Position, n (%)*		
Left	Reference	
Right	1.017 (0.513–2.084)	0.961
*EHS Classification, n (%)*		
L1-2	Reference	
L3	0.623 (0.319–1.152)	0.145
*Surgery, n (%)*		
TAPP	Reference	
TEP	0.415 (0.226–0.741)	**0.003**
*Length (cm), n (%)*		
< = 1	Reference	
< = 3	2.213 (0.501–4.904)	0.992
< = 4	2.782 (0.737–4.322)	0.992
> 4	3.717 (0.831–5.826)	0.991
*Transect, n (%)*		
No	Reference	
Yes	2.083 (1.163–3.851)	** < 0.001**

^*^OR, odd ratio; 95% CI, 95% confidence intervals; ASA, American society of anesthesiologists. Length, The length of the hernia sac,which is the distance from the deep inguinal ring to the bottom of the hernia sac

## Discussion

This study shows that transection of hernia sac is an independent factor leading to the occurrence of postoperative seroma in indirect inguinal hernia. The European Hernia Association defines and classifies seroma into type 0-IV [23]. Type 0 is no clinical seroma. Type I and II are known as incidents, which are often encountered in clinical practice and do not need to be dealt with. Types III and IV are called complications [24]. Guidelines recommend surgical treatment only for symptomatic seroma. However, patients with type III-IV seroma often need external physical therapy, and even invasive puncture may be required for diagnosis and treatment. Because this study was a retrospective study, the type of seroma was not described at that time, which was a defect of this study. For Patients whose seroma has not been absorbed for a long time, there is still the possibility of infection and secondary surgery to remove the mesh. Its main components include water, electrolyte, plasma protein and a small amount of neutrophils. Clinically, it can be diagnosed according to local symptoms, signs and imaging examination. Some surgical techniques have been reported in the literature to reduce the incidence of postoperative seromas, Such as the drainage of the preperitoneal space [25, 26], the fixation of the transverse fascia to the pectineal ligament, and the filling of the potentially dead space with fibrin glue [27], but these methods are not perfect. Aravind and Cook's study found that aseptic surgery infection was mostly secondary to postoperative seroma, and when the time was prolonged and the effusion continued to develop, severe complications such as mesh displacement, local pain, and cellulitis might occur [28]. Therefore, the incidence of seroma is a major indicator in evaluating the efficacy of laparoscopic hernia repair.

There are three possible causes of seroma. (1) Treatment of hernia sac. Patients with large hernia sac, long history, and repeated history of acute incarceration have difficulty in operation, large dissection wounds, large surgical trauma and exudation may be caused, complete reduction of hernia sac or transection of hernia sac will have an impact on the occurrence of postoperative seroma [29]. (2) Intraoperative injury. Laparoscopic surgery is different from the anterior approach in traditional surgery. When choosing the posterior approach, we should be more familiar with the anatomy of the surgical area and have a clear surgical hierarch layer to avoid excessive electrotome burns and other injuries and bleeding. Blunt separation should be used as much as possible to avoid intraoperative wound bleeding and exudation with a greater degree of distance and reduce the occurrence of seroma. (3) Selection and fixation technique of mesh. Different materials used for fixation mesh will cause foreign body and inflammatory reaction of different degrees. Compared with the weight composite mesh, the use of lightweight composite mesh can significantly reduce the incidence of postoperative seroma. The properties of the ideal mesh should prevent adhesion and promote fiber growth into the mesh [30], Furthermore, the mesh and abdominal wall were well positioned and the incidence of mesh shrinkage was reduced [31]. Patch fixation can be divided into absorbable protein glue or chemical glue fixation, and non-absorbable sutures or nail gun fixation, but its correlation with seroma remains to be further verified.

In laparoscopic hernia repair, the hernial sac approach is the most core operation steps, whether transection hernial sac is still controversial, even in the traditional inguinal hernia surgery, people are still working on the best treatment of the hernia sac [32].

In this study, there was a significant difference in the occurrence of postoperative seromas between the two groups (reduction of hernia sac group and transection of hernia sac group). Then, univariate analysis and multivariate analysis showed that the transected hernia sac could lead to postoperative seromas. Considering the baseline imbalance between the two groups, propensity score matching was carried out. After univariate analysis of the matched data of the two groups, the results still showed that transected hernia sac could lead to postoperative seroma during laparoscopic indirect inguinal hernia repair. Ruze et al. and Li, Junsheng believed that compared with complete reduction of the hernia sac, transection of hernia sac was more likely to result in higher seroma [19, 33], which was consistent with the results of this study. Some scholars believe that it would form a "curtain" closure in the inguinal canal when the hernia sac was completely reduced in an indirect inguinal hernia operation. After transection of the hernia sac in an indirect inguinal hernia operation, a residual hernia sac was formed at the distal end of the inguinal canal. The residual hernia sac was essentially parietal peritoneum and had secretion and absorption functions. Due to the obstruction of lymph reflux of the mineral part of the hernia sac, its absorption function was reduced, so its main manifestation was secretion function. Due to the anatomical position of the hernia sac, a "blind bag" effect has been formed. It is easy to collect the fluid from the preperitoneal. Combined with the results of this study, Seroma can easily be formed in a laparoscopic indirect inguinal hernia after transection of the hernia sac.

In addition, we can see that TAPP is also an independent factor in the formation of seroma. This result is consistent with Krishna A’s research [34]. TAPP has a wider surgical field, which is conducive to the surgeon's fine operation, can effectively avoid the injury of the nerve and vascular and lymphatic vessels around the hernia sac, and is more conducive to the accurate placement and fixation of the mesh. Asa is also associated with seroma formation. Patient with high ASA score are usually accompanied by basic diseases such as hypertension and hypoproteinemia, which can cause increased brittleness and permeability of microvessels and decreased osmotic pressure of plasma, and these factors also easily lead to the formation of postoperative seroma.

The occurrence of seroma after laparoscopic inguinal hernia often leads to psychological anxiety and physical pain in patients, which should be paid enough attention by Surgeons. We should fully recognize the mechanism and related factors of hematoma formation, and reduce the incidence of hematoma through various perioperative measures and intraoperative treatment of hernia sac. Patients' condition should be closely observed postoperatively, and once hematoma formation is found, active treatment should be conducted to achieve more satisfactory therapeutic effects.

## Conclusion

Transection of the hernia sac during laparoscopic indirect inguinal hernia repair can significantly lead to postoperative seroma.

## Supplementary Information

**Additional file 1: Figure S1.** Matching effect display of propensity score. After matching, the distribution of the dots is more regular than that of the unmatched control units.

## Data Availability

The data that support the findings of this study are not publicly available due to their containing information that could compromise the privacy of research participants but are available from the corresponding author upon reasonable request.

## Abbreviations

TAPP: Transabdominal preperitoneal
TEP: Totally extraperitoneal
ASA: American society of anesthesiologists
OR: Odd ratio
95% CI: 95% Confidence intervals
