# Supplementary material for: Effect of complete reduction of hernia sac and transection of hernia sac during laparoscopic indirect inguinal hernia repair on seroma
Source: BMC Surg. 2022 Apr 25;22:149. doi: 10.1186/s12893-022-01599-8 (PMC9036776; doi:10.1186/s12893-022-01599-8)
Supplement: Supplementary file 1 — Additional file 1: Figure S1. Matching effect display of propensity score. After matching, the distribution of the dots is more regular than that of the unmatched control units. [file 12893_2022_1599_MOESM1_ESM.docx]

**Figure S1** Matching effect display of propensity score. After matching, the distribution of the dots is more regular than that of the unmatched control units.

Figure S1
